# The impact of different age-friendly smart home interface styles on the interaction behavior of elderly users

**DOI:** 10.3389/fpsyg.2022.935202

**Published:** 2022-09-28

**Authors:** Chengmin Zhou, Yawen Qian, Ting Huang, Jake Kaner, Yurong Zhang

**Affiliations:** ^1^College of Furnishings and Industrial Design, Nanjing Forestry University, Nanjing, China; ^2^Jiangsu Co-innovation Center of Efficient Processing and Utilization of Forest Resources, Nanjing, China; ^3^School of Art and Design, Nottingham Trent University, Nottingham, United Kingdom

**Keywords:** elderly users, interface style, emotional interaction, eye tracking, PAD emotion scale, galvanic skin response, heart rate variability

## Abstract

Smart homes create a beneficial environment for the lives of elderly people and enhance the quality of their home lives. This study aims to explore the design of age-friendly interfaces that can meet the emotional needs of self-care elderly people from the perspective of functional realization of the operating interface. Sixteen elderly users aged fifty-five and above were selected as subjects with healthy eyes and no excessive drooping eyelids to obscure them. Four representative age-friendly applications with different interface designs were selected from the Android application market as the stimulus material for the experiment, and a total of fifteen pages were browsed independently. During the experiment, the ErgoLAB human-computer environment synchronization cloud platform was used to monitor and record the subjects' multidimensional physiological indicators of eye movements, skin electricity, and heart rate variability when using different styles of mobile application interfaces. Combined with the post-experimental PAD emotion scale data, the preferences of emotional interface design elements of the self-care elderly were analyzed to guide the subsequent design practice. The results show that: on the layout characteristics of the home page, a simple and intuitive multi-column layout or card layout combined with a bottom navigation bar type main navigation is used; on the choice of the main color, a single color with low saturation is used as the main color, with high saturation color accents to play a role in highlighting key information; on the information density of the home page, a moderate proportion of graphics and text is adopted, with low information density; on the arrangement of the page content, they try to give priority to information content with high user relevance.

## 1. Introduction

China is an aging society and the extent of aging is increasing. As society continues to develop, the demands of the new generation of older people are becoming more diverse in terms of material and spiritual life. The public's care for the elderly should not only be limited to meeting their material needs but also to researching products and services that take into account the mental health of the elderly and meet their emotional preferences.

The smart home is an important Internet of Things (IoT) use case and has a large market (Xiong et al., 2017, 2018; Hammi et al., 2022). The popularity of smart homes has led to an increasing number of consumers using smart home systems. Unlike other ICT products, older age groups are more likely than younger age groups to purchase smart home products within a specific period, and age-appropriate adaptations of smart home products will help enhance the wellbeing of older people (Shin et al., 2018). To get more people to accept smart homes, especially the older age group, it is necessary to make the interface of smart homes easy to use (Yu et al., 2022a,b). However, there is a large gap in digital device use and digital information reception among the elderly group compared to the younger group. Therefore, we need to identify the target users and the actual needs for optimization to bring a better experience for the elderly group.

The market currently lacks systematic research on user experience-centered application interface design (Zhu and Hou, 2021), and smart home age-friendly retrofits are facing multiple challenges. The interfaces of age-friendly products already on the market all have certain common problems, such as lack of versatility, high application costs for elderly users, and the need to improve the ease of use of age-friendly products. There are also some common misconceptions about aging, such as over-simplifying aging, equating aging with aging design, hoping for a single breakthrough while ignoring the bucket effect, and ignoring the long-term and enduring nature of aging needs. There is currently no clear aging-appropriate standard in the industry, and aging-appropriate has a long way to go.

To achieve active and healthy aging, it is important to consider key points such as the specific mental models and cognitive load of older people and to design products that are more comprehensive and relevant to the characteristics of older people, thus, improving the connection between older people and society. For example, when designing an interface, the visual characteristics of older people should be taken into account. Due to their reduced physical functions, older people have reduced visual abilities and focus their attention more centrally, so the interface design needs to reduce useless visual distractions (Jin, 2021). Interactive interfaces for older people should be simple and easy to navigate, with all functions and navigation visible and directly focused on the page, allowing for better and faster navigation and retrieval by older people (Balata et al., 2015). Using icons and animations that contain more pictorial information to represent objects, materials, or people in everyday life can help older people to better identify and access information, and such icons are better adapted to older people, the higher older people rate the stylistic design of such icons (Cho et al., 2015; Blaynee et al., 2016; Kreps et al., 2016; Zhou et al., 2017). Using objective eye-movement data, it was found that retrieval using flat icons tends to distract users, take longer to perform the task and increase their cognitive load compared to anthropomorphic icons (Burmistrov et al., 2015; Spiliotopoulos et al., 2018). Using older and younger users as a control group, objective experimental data and subjective scoring revealed that older people prefer anthropomorphic icons and that the use of anthropomorphic icons can effectively reduce the time spent retrieving information by older people and improve retrieval efficiency (Backhaus et al., 2018; Chen et al., 2020; Urbano et al., 2022).

Touch screen control, video control, and voice control are the three most common human-computer interaction methods in smart homes, and as the declining physical functions of older people have an impact on human-computer interaction, there is a need to design mobile application interfaces that match the behavioral abilities of older people (Tsai et al., 2017; Hou et al., 2020; Lin and Ho, 2020; Chi and Kang, 2022). With the current increased popularity of mobile phones with large screens, designers must design for the interaction of tapping the screen to achieve a balance between interactivity, usability, and user experience (Yanli et al., 2019). Related articles have found that when users swipe the smart screen, swipe directions to the right and down are more popular than swipe directions to the left and up, respectively, a finding that is consistent with subjects' left-to-right reading habits (Gao and Sun, 2015; Minakata and Beier, 2021; Liu et al., 2022). However, smartphones have a limited range of finger touch, and touch interactions may negatively affect the accuracy of responses due to being blocked by the finger, hence the need to balance the range of touch (Colley et al., 2019; Qi and Xue, 2020). Zhu et al. (2021) found that compared to other areas of the smartphone screen, users gave a significantly longer total attention time is significantly longer and the first fixation time is also mainly in the bottom area of the interface, so we can optimize the layout of the mobile application interface according to the allocation of user attention. In a visual environment with an increasing amount of available information and high information density, visual search interfaces can be optimized through techniques such as layout design, styling, and dynamic presentation to improve the efficiency of users' visual search (Gaona-Garcia et al., 2017; Simon et al., 2019; Pan et al., 2021).

In addition, for the reasonableness of the interaction interface, we need to explore and analyze the interface design of smart products for older people from the perspective of effective design. In terms of subjective measurement, Mehrabian and Epstein (1972) proposed the PAD affective state-space model based on the influence of user affective experience on design, which has been generally accepted by the psychological community. Yi et al. (2014) chose the PAD emotion scale combined with eye-movement data to measure users' effective changes when using web pages and found that between colors, pictures, elements clear and unambiguous contrasts can lead to significant enhancements in users' cognitive and behavioral abilities. By using Natural Color System (NCS) representation, Jiang found that subjects' color preference had a dominant effect on product use (Jiang and Cheung, 2020). In terms of objective measures, Calvo and Lang (2004) used eye-tracking technology to capture the number of gaze points and duration of gaze when users viewed emotional images and found that images with positive emotions attracted more attention. Liu et al. (2018) used eye-tracking experiments to study the visual highlights and visual preferences of users when viewing images. Rani et al. (2004) used wearable sensors to collect physiological signals such as Electro Dermal Activity (EDA), Heart Rate Variability (HRV), and Electromyogram (EMG) and the data was processed to detect the emotional state of the user. As electrical skin responses are more pronounced and easily accessible during the interaction, emotional responses can be well-quantified and a method for assessing emotions based on the user's physiological signals has been proposed (Mandryk et al., 2006; Mooney et al., 2006). Leite et al. (2013) measured user arousal through skin conductance when measuring user responses to the robot and found that skin electrical activity increased when the user experienced arousal, concentration, anxiety, or high cognitive load and decreased when experiencing boredom or relaxation. Kajiwara (2014) found that the mental load of the driver could be assessed using skin electrical and facial expression temperature.

From the studies that have been conducted, it can be found that appropriate interface design has a better emotional experience for smartphone use in older age groups. In the context of smartphone touch interaction, there has been little research on interface style design for older age groups. Therefore, the focus of this paper is to explore mobile product interface design that can meet the emotional needs of self-care elderly people. Four commercially available age-friendly smart home apps with different styles were selected as stimulus materials for this study. By monitoring the subjects' multidimensional physiological signal data of eye movements, electrodermal, and electrocardiogram during browsing, combined with the PAD emotion scale, the interface styles with higher user performance were explored to guide the design.

## 2. Materials and methods

### 2.1. Experimental subjects

The age, gender, and educational background of the subjects were taken into account when selecting the subjects for the experiment. Since certain tasks needed to be completed based on the content of the interface when conducting the mobile product experience, sixteen elderly people aged fifty-five and above (seven men and nine women, average age sixty-two), without color weakness or color blindness, right-handed, without physical impairment, with natural or corrected visual acuity greater than 1.0, with some schooling to be able to read and write. As the oculomotor device selected was a Tobii Fusion 250 Hz telemetric oculomotor, to facilitate the collection of oculomotor data the subjects were required to have eyes in good health and without excessive eyelid drooping to obscure them.

### 2.2. Experimental equipment

The experiment was conducted with no other people or other noise disturbances except for the instructor, the main test pair, and the subject. The room was controlled by air conditioning to maintain a certain temperature and humidity and was well lit. The equipment used for the experiment was the Ergo LAB human-machine environment synchronization cloud platform, the Tobii Pro Fusion250 eye-tracking device, and the Redmi k30 mobile phone, which was used as the carrier for the experimental interface presented to the subjects.

### 2.3. Experimental materials

(1) According to the different page layout characteristics, body-color selection, and information density of the home page of age-friendly smart APPs on the Android application market, the following four representative age-friendly APPs with different interface designs at the first level were selected as experimental materials with a total of fifteen pages, and browsing tasks were conducted for each of these four typical age-friendly mobile products.

In terms of the layout of the home page, Senior Care adopts an irregular grid layout, Smart Senior Care adopts a card layout, Senior Care Manager adopts a palace layout and C-Life Senior Care adopts a multi-column layout; in terms of the main color, the main body of Senior Care Bond is green with a variety of colors, Smart Senior Care is a high-saturation contrast color, Senior Care Manager is a high-saturation solid color and C-Life Senior Care is a low-saturation gradient color; in terms of the density of information on the home page, Senior Care Bond is denser, Smart Senior Care is denser, Senior Care Manager is the densest and C-Life Senior Care is more scattered.

(2) The experimental instructions, which are provided to the subject before the start of the experiment, include a brief description of the experimental procedure and notes on how to proceed.

(3) Affective Scale Questionnaire.

### 2.4. Experimental task design

(1) This part of the study is about the emotional experience of self-care elderly people when interacting with mobile phones, including the emotional experience of the interaction process as well as the page layout characteristics, the choice of body color, and the information density of the home page in the interface design features, the task should be designed to avoid causing excessive emotional fluctuations for the subjects. In this article, the task is autonomous browsing. By monitoring the subjects' eye movements, and electrodermal and cardiac physiological data during the browsing process, combined with the PAD emotion scale, we analyze the mobile product interface design elements that matched the emotional tendencies of the self-care elderly.

(2) The Areas of Interest (AOI) are drawn on the bottom main navigation bar of the four typical products before the experiment, the size and location of the AOI are determined according to the purpose of the study. By observing how quickly or slowly the subjects gaze at the main navigation bar during browsing, it is possible to see whether the home navigation bar can attract visual attention and whether it serves to improve the efficiency of the user's access to information.

(3) The subject enters the laboratory, is informed of the task and purpose of the experiment, and the experimenter explains the procedure and precautions to the subject.

(4) The experimenter sets up the equipment and software and adjusts the height and angle of the subject's chair to help the subject get into the correct position and posture for the experiment. The subject puts on the physiological monitoring equipment (electrodermal response, electrocardiographic response, eye tracking). The subject is then asked to sit in front of the test computer with the head held stationary and the eye tracker activated with the eyes facing the center of the screen to perform a multi-point calibration test to ensure the accuracy of the data.

(5) The subject is given a 10-min sample familiarization session to minimize performance errors due to subject inexperience.

(6) The drawn prototypes of the first-level interfaces of the four typical age-appropriate mobile products are presented on the screen of the Redmi K30Pro mobile phone.

(7) Ensure that the screen brightness and color temperature are consistent before the experiment starts. Task: Four representative mobile end products with large differences in the interface design are selected as objects for the experiment. The browsing time of each prototype interface is greater than 3 s, the experience time of each group of interfaces is variable, and users can browse at will. During the browsing process, the subjects' AOI data such as first gaze time, continuous gaze time, skin electricity, and heart rate variability are collected.

(8) After the task is completed, the subjects are asked to fill in the PAD emotion questionnaire based on their comparative feelings about using the experience.

(9) End the experiment and collate the data. Analyze the factors influencing the emotional experience in the design of age-friendly interfaces based on the experimental data and the data from the PAD Emotional Experience Scale.

### 2.5. Experimental procedure

**Figure 1:**
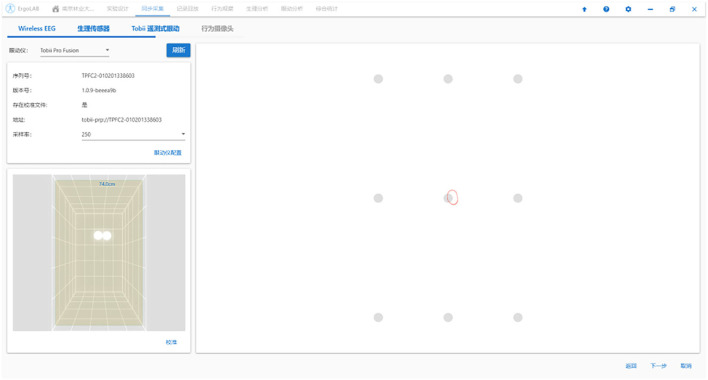
Eye movement experiment calibration page.

(1) Set up the oculomotor and the experimental stimulus materials, connect and pair the stimulus materials on the ErgoLAB cloud platform, and then enter the testing phase of each experimental instrument.

(2) Perform eye-movement calibration in the eye-movement instrument, tell the subject to keep both eyes on the stimulus material, and move the body back and forth and up and down appropriately to keep the distance between the eyes and the eye-movement instrument between 50 andnimum pupil 70 cm, perform five-point eye data calibration, if the eye-movement calibration data is not good, adjust the calibration again until it is adjusted to the best eye position, the eye-movement experiment calibration page is shown in Figure 1.

(3) Enter the instruction phase, where the subject is informed of the experimental requirements, briefly introduced to the experimental process and the operational precautions during the experiment, and answers questions about the subject.

(4) For the formal experiment, follow the pre-designed experimental procedure in Ergo LAB and carry out the browsing tasks of each typical product interface in a chaotic order, record the experimental data, and fill in the PAD emotion questionnaire after the subjects have completed all the tasks.

## 3. Experimental data processing and analysis

### 3.1. PAD scale reliability analysis

By completing the PAD emotion scale after the experiment was completed, it was possible to obtain the emotional data of sixteen subjects after completing the browsing tasks of four typical age-appropriate apps in order to analyze the differences in the emotional experience of the four products.

First, outliers were removed from the experimental data; then the Cronbach alpha coefficient test was done on the PAD scale questionnaire, as shown in Table 1, the alpha coefficient of reliability was 0.979, which is greater than 0.8 indicating that the scale reliability is very good. The scale data were tested for validity and analyzed by Kaiser-Meyer-Olkin (KMO) and Bartlett Test of Sphericity. The p-value is less than 0.01, indicating that there is a significant difference between the items, and the overall validity is indicated.

**Table 1:** Reliability coefficient test of PAD emotion scale.

**Cronbach alpha coefficient**	**Kaiser-Meyer-Olkin and Barlett Test of Sphericity**		
	**KMO values**		**0.874**
0.979		Barlett Test of Sphericity	60.888
	Barlett Test of Sphericity value	df	6
		*p*	0.000

The PAD affective scale data were then tested for normality analysis to verify that the scale data could be described by means and SDs. According to the Kolmogorov-Smirnov (K-S) test and the Shapiro-Wilk (SW) test, it is generally considered that a *p*-value greater than 0.05 is normally distributed, and the results of the tests are shown in Table 2. The results obtained from the KS test and the SW test show that each typical product of the results of the KS test and the SW test show that the PAD scale data for each typical product is greater than 0.05, so the original hypothesis cannot be rejected and the data is considered to follow a normal distribution.

**Table 2:** Normal distribution of the PAD scale of the four products.

	**Kolmogorov-Smirnov^a**			**Shapior-Wilk**		
	**Statistics**	**Degrees of freedom**	**Significance**	**Statistics**	**Degrees of freedom**	**Significance**
Senior care	0.167	12	0.200^*^	0.921	12	0.294
Smart senior care	0.187	12	0.200^*^	0.905	12	0.181
Senior care manager	0.145	12	0.200^*^	0.945	12	0.570
C-Life senior care	0.106	12	0.200^*^	0.949	12	0.615

* This is the lower limit of true saliency.

Based on the PAD scales of the four typical products completed by all subjects after completing the browsing task, the scores of pleasure (P), activation (A), and dominance (D) corresponding to each typical product were collated as shown in Table 3. The usability characteristics of the software were mapped onto the PAD emotional space model to investigate the influence of different elements of the software on the subjects' emotional experiences.

**Table 3:** Correspondence of each item and each dimension.

		**P**				**A**				**D**			
		**1**	**4**	**7**	**10**	**2**	**5**	**8**	**11**	**3**	**6**	**9**	**12**
Senior care	Score	2	−1.69	2.38	−1.75	−0.88	−0.25	1.88	−0.75	1.31	1.13	0.5	−0.94
	Average value	0.24				0				0.5			
Smart senior care	Score	2.44	−2	2.5	−1.81	−1	−0.31	0.56	−0.63	2.06	0.38	2	−1.44
	Average value	0.28				−0.35				0.75			
Senior care manager	Score	2.25	−1.63	2.56	−2.38	−1.56	−0.56	0.56	−0.13	1.69	0.56	2	−0.56
	Average value	0.2				-0.42				0.92			
C-life senior care	Score	2.25	−2.31	2.24	−2.38	−0.88	-0.88	−0.19	−0.13	0.81	0.06	0.69	−1.69
	Average value	0				−0.52				−0.03			

The Chinese version of the PAD emotion scale consists of three dimensions, P, A, and D, each with four questions, and is a semantic differential scale. When completing the scale, participants only consider the intensity of their current emotion, scoring in the direction of the adjective they prefer, with a “-4” for the word closest to the left and a “4” for the word closest to the right, and a “0” when the emotional state is in the middle, a “0” is assigned. The scores for the last three dimensions are the mean scores of the four items for that dimension, with higher mean scores indicating higher pleasantness (P) or activation (A), or dominance (D) for the corresponding dimension (Xiaoming et al., 2008).

#### 3.1.1. The effect of pleasure (P) on usability

Many studies have shown that when in a positive emotional state, the user's brain is more likely to receive and process information, making it easier to access information in the thought process and facilitate decision-making. When the usability of the test software is relatively strong and the interface design elements are appealing, it can keep the user at a good level of pleasure (P) during use, when the user can make judgments more smoothly and, thus, increase the efficiency of the operation.

#### 3.1.2. The effect of activation (A) on usability

Activation (A), also known as Arousal, refers to the different levels of total physiological activation of the organism. The correlation between activation (A) and operational performance was derived by Yerkes and student Dawson through animal experiments. It is known as the inverted U-shaped hypothesis, or the Yerkes-Dodson law (Yerkes and Dodson, 1908), because of the inverted U-shaped curve of arousal level and operational performance.

In general, user activation (D) is positively correlated with software usability, but as the level of arousal increases, the high level of mental strain during software use can lead to rapid fatigue, and also reflects the fact that users are experiencing difficulties in using the software and need to increase their activation level to think of solutions to their problems. Therefore, a positive arousal level at an appropriate and relatively low activation level is an indication of strong usability during software operation.

#### 3.1.3. The effect of dominance (D) on usability

Dominance, also known as user dominance over context and others, can be used in experiments with interface design elements to describe the degree to which users exercise personal initiative when interacting with a product.

In general, a higher degree of user dominance indicates that the user is in a more emotionally charged state when interacting with the product. This indicates a low level of user usability during the use of the software. Software with a high degree of usability enables users to use their previous usage habits to complete interaction tasks. The interaction flow of the software should be designed to match the daily behavior and usage habits of the target users, to ensure that users can operate each function module smoothly and comfortably without learning new ways of using the software and that they are always in a good emotional state during the use of the software.

### 3.2. Correlation analysis of PAD effective scale and eye movement data

First, all the oculomotor indicators obtained from the experiment were tested for normal distribution, and the results obtained showed that all the oculomotor indicators were normally distributed except for the minimum pupil diameter, the number of eye jumps, total eye jump time, and mean absolute distance indicators. Pearson's correlation analysis was carried out between the eye movement indicators of mean horizontal distance and mean vertical distance and the PAD scale data. Spearman correlations were performed on the minimum pupil diameter, the number of eye jumps, total eye jump time, and mean absolute distance with the PAD scale data.

Pearson correlation analysis, which measures the closeness between two or more variables that are correlated, requires that there is some association or probability between the variables. When the absolute value of the correlation coefficient is greater than 0.8, a strong correlation between two variables can be considered. The eye movement data obtained in this eye movement experiment such as maximum pupil diameter, mean pupil diameter, sustained gaze time, and AOI first gaze time were analyzed for correlation with P, A and D using SPSS to examine the interpretive relationship between each eye movement data and the PAD effective scale.

As seen in Table 4, the results of the bivariate Pearson test showed that maximum pupil diameter correlated with pleasure (P) and activation (A) in this oculomotor experiment, with correlation coefficients of 0.193 and 0.426, respectively, showing weak and moderate correlations. Duration of gaze was negatively correlated with activation (A), with a strong correlation coefficient of 0.964. AOI first gaze time was positively correlated with activation (A), with a strong correlation coefficient of 0.995. The rest of the data were not explanatory for any of the PAD affective categories.

**Table 4:** Correlation verification between eye movement index and PAD value.

		**Maximum pupil diameter(mm)**	**Average pupil diameter(mm)**	**Duration of gaze(s)**	**AOI first gaze time(s)**	**Number of blinks(N)**	**Average number of eye flutters(N/s)**	**Average number of blinks (N/s)**	**Average horizontal distance(px)**	**Average vertical distance(px)**
Pleasure (P)	Pearson correlation	0.193^*^	0.139^*^	−0.756	0.547	0.503	0.012	0.585	0.720	0.665
	Sig	0.008	0.006	0.244	0.453	0.497	0.988	0.415	0.280	0.335
Activation (A)	Pearson correlation	−0.426^*^	−0.492^*^	−0.964^*^	0.995^**^	−0.336	0.260	−0.192	−0.103	−0.168
	Sig	0.007	0.005	0.036	0.005	0.664	0.740	0.808	0.897	0.832
Dominance (D)	Pearson correlation	0.703	0.661	−0.34	0.117	0.853	−0.499	0.921	0.928	0.917
	Sig	0.297	0.339	0.066	0.883	0.147	0.501	0.079	0.072	0.083

* Corresponds to *p* < 0.05 and

** At the 0.01 level corresponds to *p* < 0.01, both indicate significant correlations.

Spearman correlation analysis measures the degree of dependence between two variables. In terms of a correlation coefficient, 0 indicates no correlation between the two, and the closer the correlation coefficient is to 1 or -1, the stronger the positive or negative correlation. The eye movement data obtained in this eye movement experiment, including minimum pupil diameter, number of eye jumps, total eye jump time, mean absolute distance eye movement indicators, and pleasure (P) activation (A) and dominance (D), were analyzed using SPSS to test the correlation between each eye movement data and the PAD effective scale.

As seen in Table 5, Spearman's Rho revealed that the number of eye jumps, total eye jump time, and mean absolute distance was positively correlated with dominance (D) with a correlation coefficient of 1, which was a strong correlation.

**Table 5:** Eye movement index and PAD value Spearman correlation test.

		**Minimum pupil diameter (mm)**	**Number of eye jumps (N)**	**Total eye jump time (s)**	**Average absolute distance (px)**
Pleasure (P)	Spearman correlation coefficient	-0.600	0.400	0.400	0.400
	Sig	0.400	0.600	0.600	0.600
Activation (A)	Spearman correlation coefficient	-0.800	0.200	0.200	0.200
	Sig	0.200	0.800	0.800	0.800
Dominance (D)	Spearman correlation coefficient	0.400	1.000^**^	1.000^*^	1.000^*^
	Sig				

* Corresponds to *p* < 0.05;

** Correlation significant at 0.01 level (two-tailed).

The correlation between maximum pupil diameter and mean pupil diameter and the PAD affective scale was tested to be consistent with previous findings (Yi et al., 2014), and the Statistical Product Service Solutions (SPSS) correlation analysis demonstrated that the duration of gaze, AOI first gaze time, number of eye jumps, total eye jump time, and mean absolute distance could also explain the changes in the PAD affective values, so it was possible to combine the maximum pupil Eye movement indicators such as maximum pupil diameter, mean pupil diameter, AOI first gaze time, and sustained gaze time can be combined with the PAD scale to analyze the eye movement data of the interaction tasks of four typical products from a combined perceptual and rational perspective to derive the interface design elements of an age-friendly emotional interaction service platform.

## 4. Results

### 4.1. Eye movement data analysis

**Figure 2:**
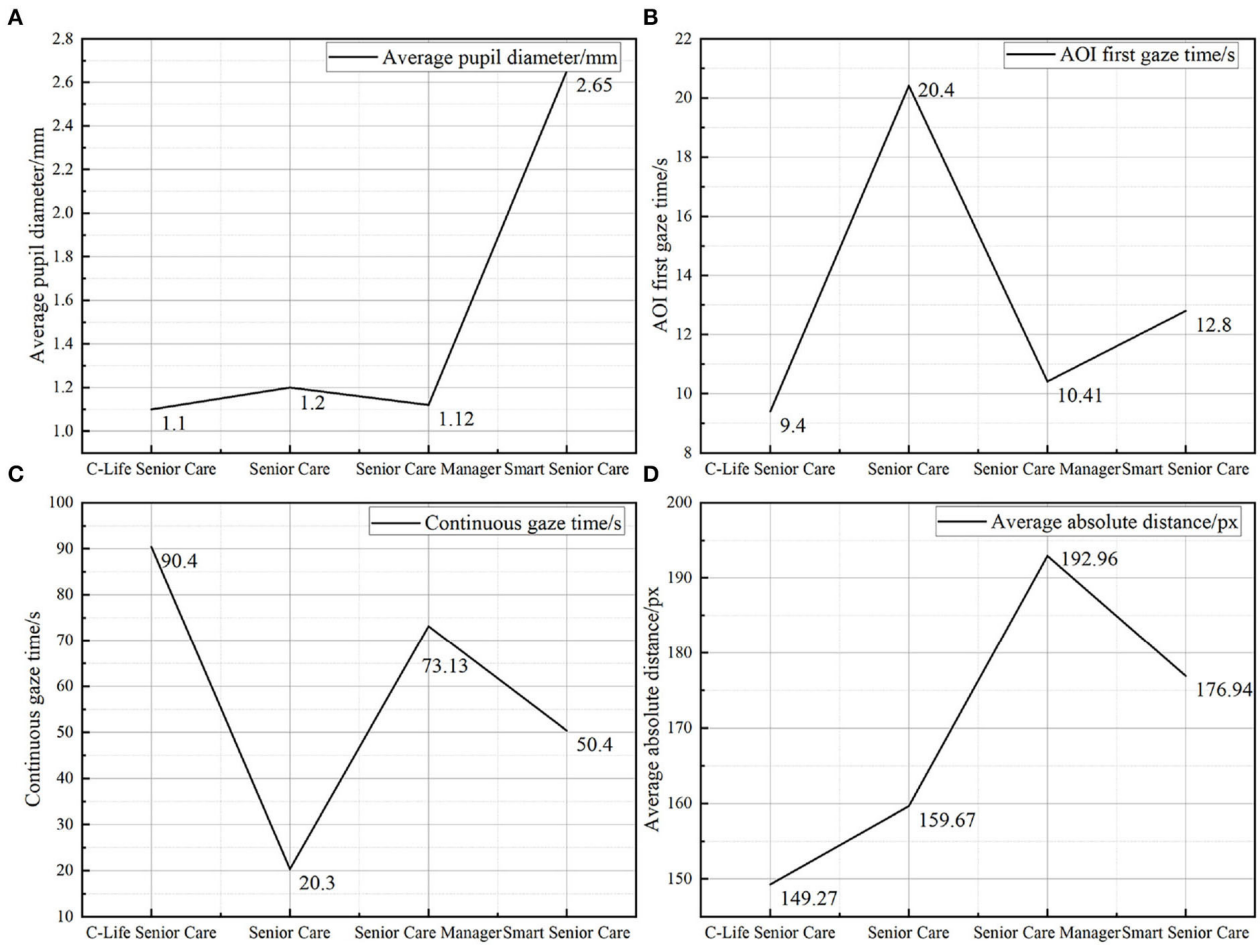
Eye movement data. Panel **(A)** is the average pupil diameter of the four products, panel **(B)** is the first gaze time of AOI of four products, panel **(C)** is the constant gaze time of four products, and panel **(D)** is the average absolute distance of the four products.

According to the correlation analysis between the eye movement data and the PAD emotion scale, the pupil maximum and pupil mean were positively correlated with pleasure (P) and negatively correlated with activation (A); AOI first gaze time was positively correlated with activation; continuous gaze time was negatively correlated with activation; the number of eye jumps, total eye jump time and mean absolute distance were all positively correlated with dominance. Therefore, the average pupil diameter, AOI first gaze time, sustained gaze time, and average absolute distance were selected to analyze the interface design elements that affect the user experience of the four products, as shown in Figure 2.

#### 4.1.1. Average pupil diameter analysis

As the average pupil diameter is positively correlated with Pleasure (P) and negatively correlated with Activation (A), Figure 2A shows that the average pupil diameter data for Smart Senior Care is much higher than the other three products, and the interface design elements reflect a card-based design with a clear layout, moderate information density, and intuitive color contrast. The user does not need to overthink and increases their own activation level to access and process information to complete the interaction process and is able to have a higher level of pleasure (P), which is also consistent with the pleasure ranking measured by the PAD emotion scale.

#### 4.1.2. AOI first gaze time analysis

The AOI was drawn on the main navigation bar because the time that the user attends to the main navigation bar determines whether the user can quickly and accurately find useful information to complete the interaction process when they are at a low level of activation (A).

It is known that the first gaze time of AOI is positively correlated with the activation level, and the earlier the first gaze time, the higher the activation level. The first gaze time of the AOI is shown in Figure 2B, and it is found that the first gaze time of the AOI of C-Life Senior Care is earlier than that of Senior Care Manager and earlier than that of Smart Senior Care and earlier than that of Senior Care, they were 9.4, 10.41, 12.8, and 20.4 s, respectively, with the Senior Care having the latest AOI first gaze.AOI first look time is positively correlated with activation level (A), and correspondingly, the activation level (A) level is higher in Senior Care, which also proves that the user's neurophysiological activation level is higher during the interaction task in Senior Care. This is due to the fact that the navigation icons on the home page of Senior Care are inherently small and dense with information, and the irregular grid format layout leads to a lot of information and clutter on the page, which requires a higher activation level to process the graphic information, which hinders the efficiency of users in accessing the main navigation information, hence the late first look of the AOI. When designing the interface of an emotional interaction service platform for the elderly, we should try to avoid setting up too much information on the homepage, so that the elderly can complete the interaction process with a low activation level (A) and, thus, have a more pleasant emotional experience.

#### 4.1.3. AOI sustained gaze duration analysis

It is known that the sustained gaze time is negatively correlated with the activation degree (A), and the sustained gaze time of the stimulus material is counted as in Figure 2C, C-Life Senior Care has the longest sustained gaze time, followed by Senior Care Manager, then Smart Senior Care and the shortest gaze time is Senior Care, which shows that the highest activation degree (A) is Senior Care, and the lowest is C-Life Senior Care.

According to the data compiled from the gaze duration and the average value of each app, it can be seen that product interfaces with a simple layout and less use of color in the stimulus material, such as C-Life Senior Care and Senior Care Manager, receive relatively longer gaze instead. This is because a dense arrangement of text can make the page appear visually constricted, creating a sense of internal tension and oppression, which affects the user's dominance (D) and activation (A). The higher the level of dominance (D) and activation (A), the more the user is in a state of non-relaxation. For example, the overall layout of the Senior Care page is dense, with more content and more text, which hinders the user from processing the main navigation information and the focus point is scattered, which is not conducive to forming a long-lasting gaze for effective information, resulting in a higher activation (A) and dominance (D), which has a certain impact on the user's pleasure (P), therefore, in The average duration of gaze is the shortest among the four apps.

#### 4.1.4. AOI means absolute distance analysis

It is known that the number of eye jumps, the total eye jump time, and the average absolute distance are all positively correlated with the dominance degree (D). A line graph of the average absolute distance is drawn for the four typical products, as shown in Figure 2D, which shows that the average absolute distance of the four products is 192.96, 176.94, 159.67, and 149.27, respectively. The Senior Care Manager has the highest average absolute distance and, therefore, the highest dominance (D) The interface design of the Senior Care Manager is characterized by a palace format layout, high purity color blocks, and high information density. The higher color saturation of the page is more likely to attract the user's attention and dominate the user's visual trajectory, while the higher information density also leads to a higher dominance (D) level because it puts more pressure on the user to process information.

In general, the shorter the AOI first gaze time, the clearer the interaction process and the lower the difficulty of processing information on the home page, which is reflected in the low density of information on the home page, clear page layout, and low overall color saturation, so the interaction task can be completed by habitual gestures and reading, showing a low activation (A) and high pleasure (P); the longer the continuous gaze time on the page, the higher the user is attracted to the page. For example, in the interface design of C-Life Senior Care, although it follows the interface design principles of low information density on the homepage, clear multi-column layout and low saturation, the overall Pleasure level (P) is not high. According to the follow-up interviews, the subjects did not obtain effective information related to themselves after paying attention to the health data in the interface information during the experiment, thus the interface design was reasonable but the content was not sufficient, which had a greater impact on the user's pleasure (P).

### 4.2. Analysis of electrical skin signal data

The electrical skin response is one of the most sensitive forms of emotional feedback, originating from the voluntary activation of the skin's sweat glands. Closely related to mood, arousal, and attention, it is the most widely used type of measure in physiological response systems.

**Figure 3:**
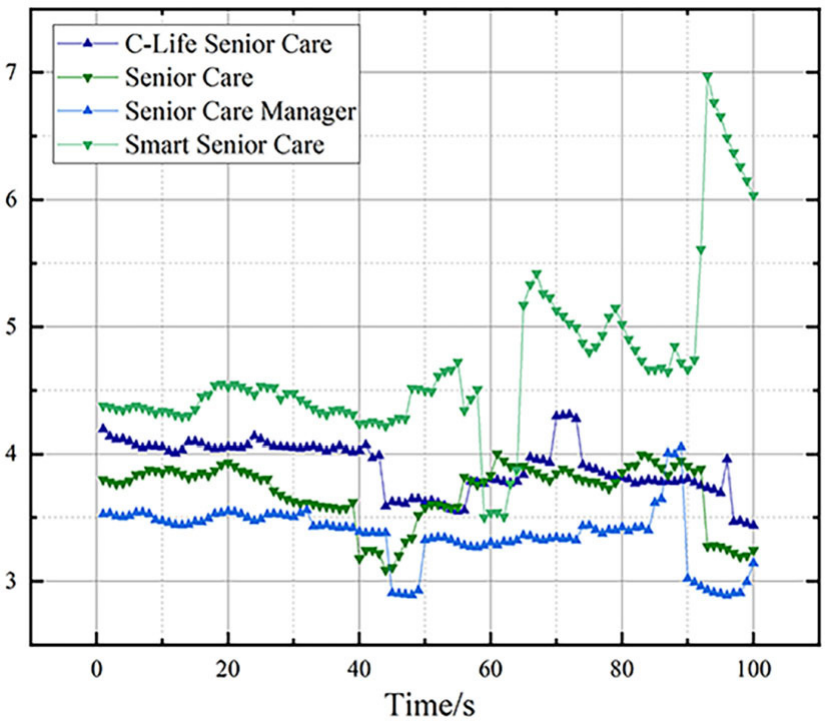
The average value of the skin signal movement of the four products.

The dermal electrical signals recorded on the cloud platform were analyzed after noise reduction processing, and the dermal electrical signal line graph was caused by the fluctuation of the subjects' emotions while using the four typical products. The electrodermal levels were relatively higher when the user was active and lower when relaxed, and the higher the amplitude of the physiological data, the higher their activity level. The results are shown in Figure 3, where the overall level of skin electricity is higher for Smart Senior Care than for the other three products. This is due to the fact that Smart Senior Care's overall page design is well-designed and easy to operate, making it easier to attract the user's attention when they are most active. As the browsing content changed and the browsing time continued, the user's skin electrical response began to fluctuate, with the fold gradually dropping, at which point attention was distracted or even most likely in unconscious behavior, with the skin electrical signal of Smart Senior Care also dropping significantly later than the other three products, which is consistent with the conclusions drawn from the eye-movement combined with the subjective scale above.

### 4.3. Analysis of heart rate variability data

Heart Rate Variability (HRV) is the small increase or decrease in an individual's instantaneous heart rate between successive heartbeats. The level of HRV can be considered an indicator of physiological stress or arousal, with increased arousal at lower HRV and decreased arousal at higher HRV. High HRV represents better stress tolerance, while low HRV implies a risk of anxiety and depression. HRV can be reduced in times of stress and mood swings, so changes in HRV can be analyzed to reveal the user's psychological response to different stimuli.

**Figure 4:**
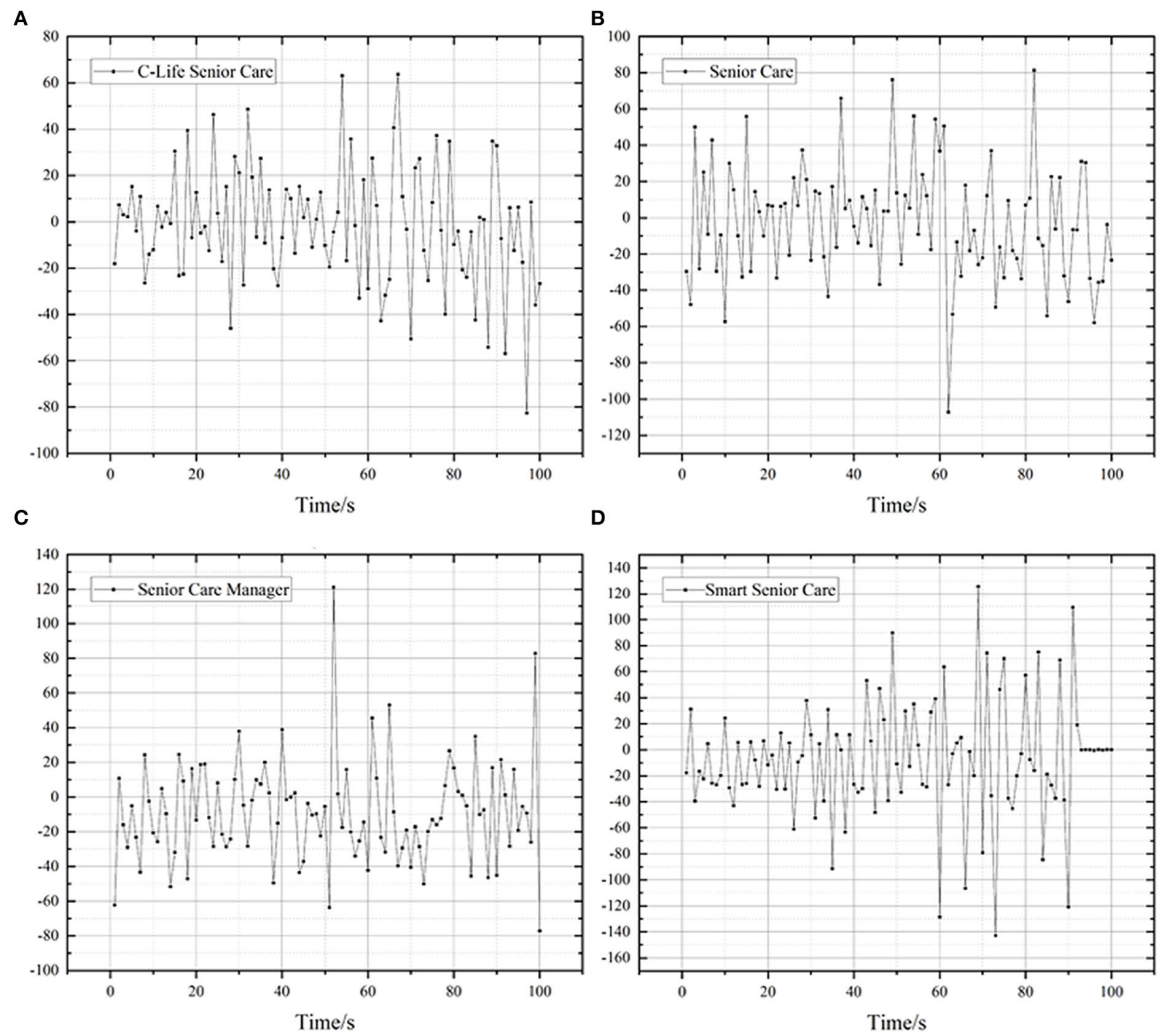
Mean heart rate variability of the four products. **(A)** C-Life Senior Care, **(B)** Senior Care, **(C)** Senior Care Manager, **(D)** Smart Senior Care.

The mean values of heart rate variability when subjects used the four typical products were extracted and compared to explore the pattern of heart rate variability when subjects received stimuli. The results of the analysis are shown in Figure 4. As the browsing time was extended, the heart rate variability of Smart Senior Care was greater than the heart rate variability of the other three products, when the user's parasympathetic activity was greater and dominated the mode, indicating that the user was better adapted to Smart Senior Care and was in a relaxed state. Senior Care Manager is not conducive to content recognition and access in this visual environment due to its high page information density, which tends to lead to users being in an unrelaxed state with overall low heart rate variability values. This result remains consistent with the conclusions drawn above.

## 5. Conclusion

Based on the above analysis of the combination of eye movement data with PAD scale data and electrodermal and electrocardiographic data, the interface design elements of the age-friendly emotional interaction service platform are summarized in the following four aspects.

For example, the irregular grid layout combines multiple layouts on a single page, and when there are too many graphics and text information, it is not easy to focus on the information, and it is easy to produce a “boredom” mentality and a dominated emotional experience, which has a relatively low dominance (D) is low and the pleasure level (P) is low. Card-based layouts and multi-column layouts are simpler and more intuitive, making it easier for users to access effective information directly. The use of a clear and intuitive multi-column layout or card layout combined with a bottom navigation bar makes it easier for the user to complete the interaction task, reduces activation (A), and brings a better emotional experience.

In terms of subject color selection, page color has an impact on dominance (D). Color elements can attract users' visual attention, and clear color changes and intuitive color contrasts can effectively divert the user's eyes and play a guiding role in the user's vision, making the user's attention focused or diverted. The right choice of color can strengthen the user's grasp of the key information on the page, while an inappropriate choice of color can affect the user's independent interaction habits, resulting in an overly dominant interface (D), which makes it difficult for the user to effectively access information and is in a state of low pleasure (P). The main color of the interface design should be a single color with low saturation, with a high saturation of color embellishment, to highlight the key information.

In terms of the density of information on the home page, based on the length of time spent looking at each typical mobile product page and the average of the data collated, it can be seen that the product interfaces with less information density on the home page, such as C-Life Senior Care and Senior Care Manager, are viewed for a longer period of time. This is because the dense arrangement of images and text makes the overall visual effect of the page appear constricted, creating a sense of internal tension and oppression, which affects the user's perception of pleasure (P), dominance (D), and activation (A). When the density of graphic information is moderate, users are able to access and process information quickly and effectively and are not “bored” by the need to deal with complex and large textual information, and the level of pleasure (P) is relatively high.

The content of the page is organized in such a way as to ensure relevance to the user. For example, C-Life Senior Care follows the interface design principles of low information density on the homepage, clear multi-column layout, and low overall saturation, but the Pleasure Level (P) is not high. According to the follow-up interviews, the subjects did not obtain any personal information after paying attention to the health data in the interface, so the interface design was reasonable but the content was not sufficient, which had a greater impact on the user's pleasure (P).

## 6. Discussion

The experimental procedure was first determined by monitoring the subjects' eye movement, electrocortical and electrocardiographic data during the users' completion of the browsing task and filling in the PAD affective scale after the completion of the experimental task. SPSS analysis was used to verify the reliability of the PAD scale and the normality of the PAD scale and the eye movement indicators, and correlation analysis was conducted between the eye movement indicators and the corresponding PAD scale. According to the Pearson correlation test, it was obtained that the maximum pupil diameter was correlated with pleasure (P) and activation (A) in this eye movement experiment, and the correlation coefficients were 0.193 and 0.426 respectively, which were weak and moderately strong correlations. Duration of gaze was negatively correlated with activation (A) with a strong correlation coefficient of 0.964, while AOI first gaze time was positively correlated with activation (A) with a strong correlation coefficient of 0.995. The number of eye jumps, total eye jump time, and mean absolute distance was positively correlated with dominance (D) with a correlation coefficient of 1, a strong correlation according to the Spearman correlation test.

Visual graphical analysis, as well as data analysis of the four selected age-appropriate products, were carried out based on a combination of emotional and rational approaches. The design of the interface of the emotional interaction service platform for self-care elderly was obtained: on the layout of the home page, a simple and intuitive multi-column layout or card layout was adopted, combined with a navigation bar at the bottom as the main navigation method; on the choice of the main color, a single color with low saturation was used as the main color, with a high saturation color embellishment to highlight the key information; on the information density of the home page, a moderate proportion of graphics and text was adopted, with low information in the home page information density, take the form of a moderate proportion of graphics and low density of information; in the arrangement of page content, try to give priority to information content with a high degree of relevance to users.

The limitations of this article should also be noted. First of all, for the existing mobile age-friendly products in the market, only four apps with a wide range of design styles have been selected as stimulus material for this paper, and selecting more age-friendly products or giving more complex operational tasks may differ from the current results. The sample data selected in this paper is limited, and users can choose the posture according to their comfort and the actual situation of the environment when using mobile products, more samples should be added in the future to verify the findings of this article. The elderly population is affected by physical function, and the size of the mobile phone screen, the different positions and shapes of the device buttons, finger dexterity, and the reaction time of the subjects all affect the accuracy of the users' operations (Grant, 2020; Sanchez et al., 2021). In addition, the electrical skin response and heart rate can vary depending on individual differences, level of skin dryness, and experimental environment. Therefore, other potential factors affecting the use of smart screens by older users should be explored in subsequent studies.

## Data availability statement

The raw data supporting the conclusions of this article will be made available by the authors, without undue reservation.

## Ethics statement

Written informed consent was obtained from the individual(s) for the publication of any potentially identifiable images or data included in this article.

## Author contributions

CZ contributed to the framework ideas and experimental design. YQ and YZ helped with the experimental manipulation and the collection and analysis of raw data. TH plotted the graphs. JK provided guidance on language improvement and article layout.

## Funding

Part of this study was supported by the National Key Research and Development Program (2017YFD0601104), part of it was supported by the 2020 Jiangsu Postgraduate International Smart Health Furniture Design and Engineering project, and Nanjing Forestry University Ecological Health Home Furnishing Industry-University-Research International Cooperation Joint Support for laboratory projects sponsored by Qing Lan Project.

## Conflict of interest

The authors declare that the research was conducted in the absence of any commercial or financial relationships that could be construed as a potential conflict of interest.

## Publisher's note

All claims expressed in this article are solely those of the authors and do not necessarily represent those of their affiliated organizations, or those of the publisher, the editors and the reviewers. Any product that may be evaluated in this article, or claim that may be made by its manufacturer, is not guaranteed or endorsed by the publisher.
